# Imaging of IPMN of the pancreas: evaluation of malignant potential and resectability

**DOI:** 10.1186/1470-7330-15-S1-O6

**Published:** 2015-10-02

**Authors:** Jeong Min Lee

**Affiliations:** 1 Department of Radiology, Seoul National University Hospital, Seoul 110-744, South Korea

## 

Intraductal papillary mucinous neoplasm (IPMN) of the pancreas is defined as a tumor growing in the main duct or branch duct of the pancreas, with differentiated papillary features and production of atypical mucin, as well as segmental or diffuse dilation of the main pancreatic duct (MPD), cystic dilation of the secondary branches, or both. Histologically they exhibit a wide spectrum of dysplastic changes from low and moderate grade dysplasia to high grade dysplasia (in situ carcinoma) to eventually, invasive carcinoma. The international consensus guidelines 2012 for the management of IPMNs and mucinous cystic neoplasms of the pancreas have been issued. The new international consensus guidelines recommend multidetector row CT or MR imaging with MR cholangiopancreatography (CP) in the evaluation of pancreatic cysts larger than 1 cm to check for high-risk stigmata or worrisome features. In cysts that show high-risk stigmata, surgical management is recommended. In this lecture, I would like to discuss the diagnostic performance of magnetic resonance imaging (MRI) with MR cholangiopancreatography (MRCP) and diffusion weighted imaging, and MDCT in determining the malignant potential and surgical resectability of pancreas IPMNs using pathologic and surgical analyses as reference standards. In addition, I would like to discuss interobserver agreement of each diagnostic criterion on CT and MRI for evaluation of the malignant potential of IPMNs.
